# Extensive genetic diversity of Rickettsiales bacteria in multiple mosquito species

**DOI:** 10.1038/srep38770

**Published:** 2016-12-09

**Authors:** Wen-Ping Guo, Jun-Hua Tian, Xian-Dan Lin, Xue-Bing Ni, Xiao-Ping Chen, Yong Liao, Si-Yuan Yang, J. Stephen Dumler, Edward C. Holmes, Yong-Zhen Zhang

**Affiliations:** 1 State Key Laboratory of Infectious Disease Prevention and Control, Department of Zoonoses, National Institute for Communicable Disease Control and Prevention, Chinese Center for Disease Control and Prevention, Beijing; Collaborative Innovation Center for Diagnosis and Treatment of Infectious Diseases, Hangzhou, China; 2 Wuhan Center for Disease Control and Prevention, Wuhan, Hubei Province, China; 3 Wenzhou Center for Disease Control and Prevention, Wenzhou, Zhejiang Province, China; 4 Ganzhou Center for Disease Control and Prevention, Ganzhou, Jiangxi Province, China; 5 Department of Pathology, Uniformed Services University for the Health Sciences, Bethesda, MD 20814, USA; 6 Marie Bashir Institute for Infectious Diseases and Biosecurity, Charles Perkins Centre, School of Life and Environmental Sciences and Sydney Medical School, The University of Sydney, Sydney, NSW 2006, Australia

## Abstract

Rickettsiales are important zoonotic pathogens, causing severe disease in humans globally. Although mosquitoes are an important vector for diverse pathogens, with the exception of members of the genus *Wolbachia* little is known about their role in the transmission of Rickettsiales. Herein, Rickettsiales were identified by PCR in five species of mosquitoes (*Anopheles sinensis, Armigeres subalbatus, Aedes albopictus, Culex quinquefasciatus* and *Cu. tritaeniorhynchus*) collected from three Chinese provinces during 2014–2015. Subsequent phylogenetic analyses of the *rrs, groEL* and *gltA* genes revealed the presence of *Anaplasma, Ehrlichia, Candidatus* Neoehrlichia, and *Rickettsia* bacteria in mosquitoes, comprising nine documented and five tentative species bacteria, as well as three symbionts/endosybionts. In addition, bacteria were identified in mosquito eggs, larvae, and pupae sampled from aquatic environments. Hence, these data suggest that Rickettsiales circulate widely in mosquitoes in nature. Also of note was that *Ehrlichia* and *Rickettsia* bacteria were detected in each life stage of laboratory cultured mosquitoes, suggesting that Rickettsiales may be maintained in mosquitoes through both transstadial and transovarial transmission. In sum, these data indicate that mosquitoes may have played an important role in the transmission and evolution of Rickettsiales in nature.

The order Rickettsiales (*Alphaproteobacteria*) comprises of a group of obligate intracellular bacteria that are common parasites of eukaryotes. The order contains three documented families (*Anaplasmataceae, Holosporaceae*, and *Rickettsiaceae*) as well as one tentative family (*Candidatus Midichloriaceae*)^1^,^2^. Rickettsiales are well known as zoonotic pathogens, causing such severe human diseases as anaplasmosis, ehrlichiosis, rickettsioses, and scrub typhus^3^, as well as being associated with extensive agricultural losses^4^,^5^. The global incidence and geographic range of rickettsial diseases is seemingly experiencing its second pronounced increase in the last 40 years^6^,^7^, and the incidence of human monocytotropic ehrlichiosis (HME) and human granulocytic anaplasmosis (HGA) have increased steadily since their discovery in the 1980s and 1990s, respectively^8^,^11^. Due to better diagnostic techniques and enhanced surveillance, the identification of new Rickettsiales and/or their associated diseases has increased markedly over the last 10 years^7^,^12^,^13^,^14^,^15^, and bacteria that had previously been considered nonpathogenic to humans are now associated with disease^6^,^7^,^14^,^15^,^16^. Clearly, Rickettsiales will present a considerable public health challenge for the forseeable future.

One of the most striking features of Rickettsiales is their diverse host range that includes protists, hydra, annelids, arthropods, vertebrates, and even plants^15^,^17^,^18^,^19^,^20^. However, only ticks have been found to act as the vectors for bacteria of the genera *Anaplasma* and *Ehrlichia*^3^,^14^,^18^. In nature, *Rickettsia* bacteria are spread by either transstadial and transovarial (i.e. vertical) transmission in their arthropod hosts, or by horizontal (co-feeding) transmission through an infected vertebrate^15^,^17^,^21^,^22^. To date, although *Anaplasma* and *Ehrlichia* bacteria are passed transstadially in ticks, definitive evidence for transovarial transmission is not yet available^3^,^14^, such that vertebrates (e.g. rodents and ruminants) are required as their main amplifying hosts.

Mosquitoes are members of the family *Culicidae* and comprise more than 3,500 species. Many species (although only females) feed on blood from various vertebrate hosts, including mammals and birds. Mosquitoes are the most important vector of disease-causing pathogens in humans and animals^23^, with, for example, more than one-half of the global population at risk for mosquito-borne infections such as dengue and malaria^24^,^25^. However, despite their significance in pathogen transmission, with the sole exception of the genus *Wolbachia*^24^,^26^, little is known about the circulation and transmission of Rickettsiales bacteria in mosquitoes. Indeed, there is only a single description of the 16 S rRNA (*rrs*) gene of *Anaplasma* bacteria in the midgut of *Anopheles* mosquitoes, although it could not be excluded that the bacteria were from ingested blood^27^, while a recent experimental study reported the transmission potential of *R. felis* by *Anopheles gambiae* mosquitoes^28^.

To determine whether mosquitoes are indeed hosts of Rickettsiales bacteria, we collected mosquitoes at different life stages – adults, eggs, larvae, and pupae – from a variety of locations in Hubei, Jiangxi, and Zhejiang provinces, China, and examined the prevalence of *Anaplasma, Ehrlichia, Candidatus* Neoehrlichia, and *Rickettsia* bacteria. In addition, we conducted a laboratory investigation of the potential mosquito transmission of Rickettsiales.

## Results

### Collection of mosquitoes and detection of Rickettsiales DNA

During 2014–2015, 971 adult mosquitoes were collected from three regions in China: (i) Wuhan city in Hubei province, (ii) Yudu county in Jiangxi province, and (iii) Cixi city in Zhejiang province (see Supplementary Fig. S1). The numbers, species, and geographic distributions of the adult mosquitoes collected are shown in Table 1. After morphological examination and sequence analysis of the 18S rRNA gene, *Anopheles sinensis, Armigeres subalbatus, Aedes albopictus, Culex quinquefasciatus* and *Cu. tritaeniorhynchus* mosquitoes were identified. In addition, approximately 950 eggs, 528 larvae, and 554 pupae of these mosquitoes were collected from aquatic environments in Wuhan city (Table 2). The eggs were pooled into groups of approximately 19 according to species.

PCR products of the expected size for the *rrs* gene were successfully amplified from the adult and juvenile mosquitoes collected in the field and reared in the laboratory (Tables 1, 2, 3, [Table t2], [Table t2], [Table t3], see Supplementary Table S1). Genetic analyses of these bacterial *rrs* gene sequences revealed that all the bacteria identified in mosquitoes belonged to the order Rickettsiales (see below) and exhibited high similarity in the *rrs* gene with those of the *Anaplasma, Ehrlichia, Candidatus* Neoehrlichia, and *Rickettsia* genera, with percentage identities greater than 95.6%, 97.6%, 99.0%, and 95.2%, respectively. Hence, these data clearly reveal that multiple generations of Rickettsiales naturally co-circulate in both adult and juvenile mosquitoes.

### Phylogenetic analysis of Rickettsiales gene sequences recovered from mosquitoes

**Figure 1:**
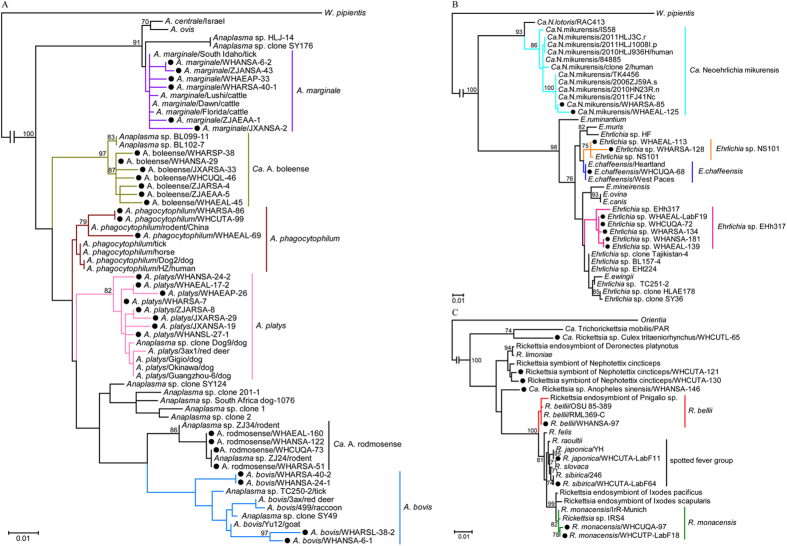
Phylogenetic trees of Rickettsiales *rrs* gene sequences. Phylogenetic trees for bacteria of the genera *Anaplasma* (**A**), *Ehrlichia* and *Candidatus Neoehrlichia* (**B**), and *Rickettsia* (**C**). Numbers at each node indicate bootstrap values. Trees (**A–C**) were mid-point rooted for clarity and the scale bar represents the number of nucleotide substitutions per site. Lineages shown in purple in panel A and taxa marked by circles in panels A–C depict sequences obtained in this study.

Phylogenetic analysis of the *rrs* gene sequences revealed that the Chinese mosquitoes collected in this study contained bacteria from the *Anaplasma, Ehrlichia,* or *Candidatus* Neoehrlichia genera of the family *Anaplasmataceae*, and from the genus *Rickettsia* of the family *Rickettsiaceae* (Fig. 1). Within the genus *Anaplasma*, the *rrs* gene sequences fell into six clusters on the phylogeny (Fig. 1A), corresponding to (i) four documented species: *A. bovis, A. marginale, A. phagocytophilum*, and *A. platys*, and (ii) two novel candidate species: *Anaplasma* sp. Bole (designed as *Candidatus* Anaplasma boleense) and *Anaplasma* sp. rodmos (designed as *Candidatus* Anaplasma rodmosense). *Candidatus* A. boleense bacteria, which were first identified in ticks sampled from Bole in the Xinjiang Uygur Autonomous Region, China^18^, formed two distinct lineages according to their vector origins. It is notable that the mosquito *Candidatus* A. rodmosense bacteria were closely related to *Anaplasma* sp. ZJ24 identified in *Rattus losea* rat from Zhejiang (FJ182047). Within the clusters of *A. bovis*, and *A. platys* bacteria, the mosquito *Anaplasma* bacteria were clearly distinct from those described previously (with 0.6–2.9% and 0.4–2.1% differences, respectively). *A. bovis* bacteria formed two lineages, one of which was most closely related to an *A. bovis* isolated from goats in China^29^. Particularly notable was that the *A. platys* bacteria from mosquitoes were diverse, showing 97.9–99.6% identity with known *A. platys,* and occupied the basal position in this cluster. *A. phagocytophilum* bacteria were also distinct but most closely related to a strain isolated from a *R. norvegicus* rat^30^. Finally, *A. marginale* bacteria were closely related to a cattle isolate sampled in Henan province^31^, which adjoins Hubei province, as well as those sampled from cattle in Australia and the USA.

The *Ehrlichia* bacteria from mosquitoes formed three clusters, compatible with the existence of three species (Fig. 1B). One cluster comprised the mosquito *rrs* gene sequences WHARSA-128 and HHAEAL-113 and *Ehrlichia* sp. NS101 identified from deer in Japan. In contrast, the mosquito *rrs* gene sequence WHCUPA-68 was more closely related to those of human *E. chaffeensis*^32^. The remaining mosquito *Ehrlichia* bacteria formed a distinct cluster most closely related to the tick bacteria *Ehrlichia* sp. EHh317. *Candidatus* Neoehrlichia mikurensis bacteria were also identified in mosquitoes, showing a close relationship with those previously identified in rodents and humans^33^.

The *Rickettsia* bacteria sampled from the mosquitoes in this study were closely related to *R. monacensis* (sequence WHCUQA-97), *R. bellii* (WHANSA-97), *R. japonica* (WHCUTA-LabF11), *R. sibirica* (WHCUTA-Lab64), respectively (Fig. 1C). The sequences WHCUTA-121 and WHCUTA-130 exhibited a close evolutionary relationship with those of the *Rickettsia* symbiont of *Nephotettix cincticeps*^34^. Interestingly, the sequence of WHCUTL-65 was distinct, but most closely related to Candidatus Trichorickettsia mobilis isolated from *Paramecium nephridiatum* (97.0% identity)^35^, while the sequence WHANSA-146 was distinct from all known *Rickettsial* bacteria. Hence, our data suggest the presence of two novel *Rickettsia* species (or symbionts/endosybionts) in mosquitoes, designated as *Candidatus* Rickettsia sp. Anopheles sinensis and *Candidatus* Rickettsia sp. Culex tritaeniorhynchus to reflect their host species.

We also attempted to amplify other two genes – citrate synthase (*gltA)* and heat shock protein (*groEL*) – from all Rickettsiales DNA-positive mosquitoes (see Supplementary Table S1). However, compared to the *rrs* gene, these gene sequences were difficult to amplify from mosquito samples. Consequently, *groEL* gene sequences were only recovered from some samples positive for *Anaplasma, Ehrlichia*, and *Rickettsia*, while *gltA* gene sequences were obtained only from some samples positive for *Anaplasma* and *Rickettsia*.

**Figure 2:**
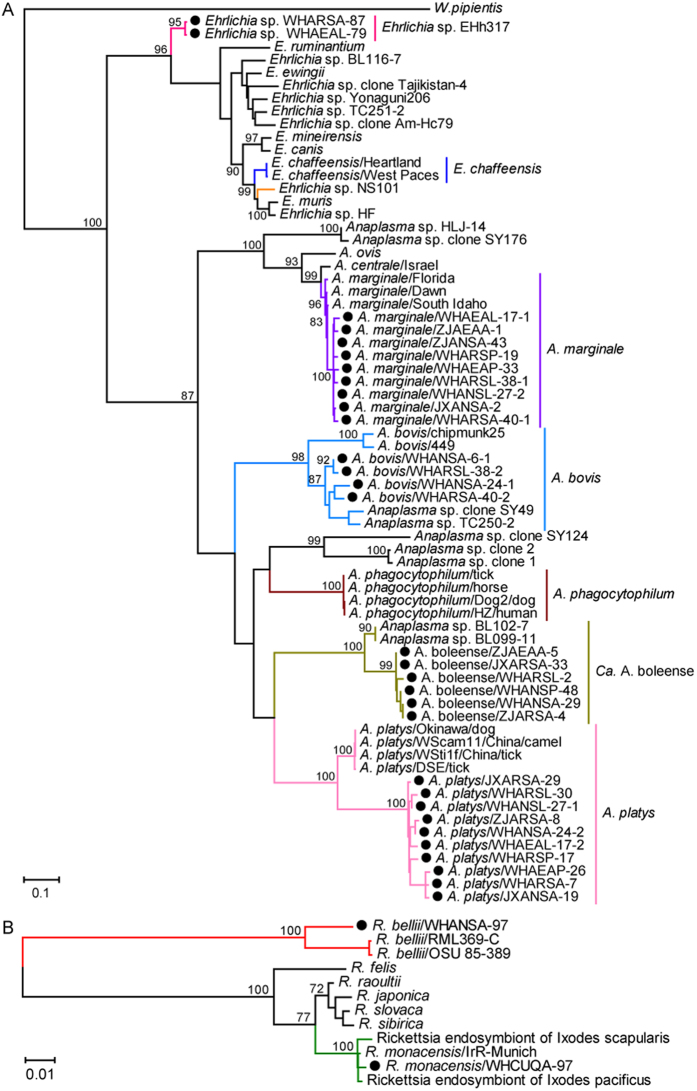
Phylogenetic trees of the heat shock protein gene (*groEL*) of bacteria of the genera *Anaplasma* and *Ehrlichia* (**A**) and *Rickettsia* (**B**). The figure description follows that of Fig. 1.

In the *groEL* gene tree (Fig. 2A), all sequences recovered from *Anaplasma* mosquitoes fell into four clusters corresponding to *Candidatus* *A. boleense, A. bovis, A. marginale*, and *A. platys*. Notably, within each of the clusters, the sequences from mosquitoes formed distinct lineages similar to those observed for *rrs* gene. The *groEL* gene sequences recovered from the *Ehrlichia* positive samples were also closely related each other and formed a distinct cluster. Finally, those mosquito sequences recovered from *Rickettsia* positive samples were closely related to those from *R. belli* and R. monacensis (Fig. 2B).

**Figure 3:**
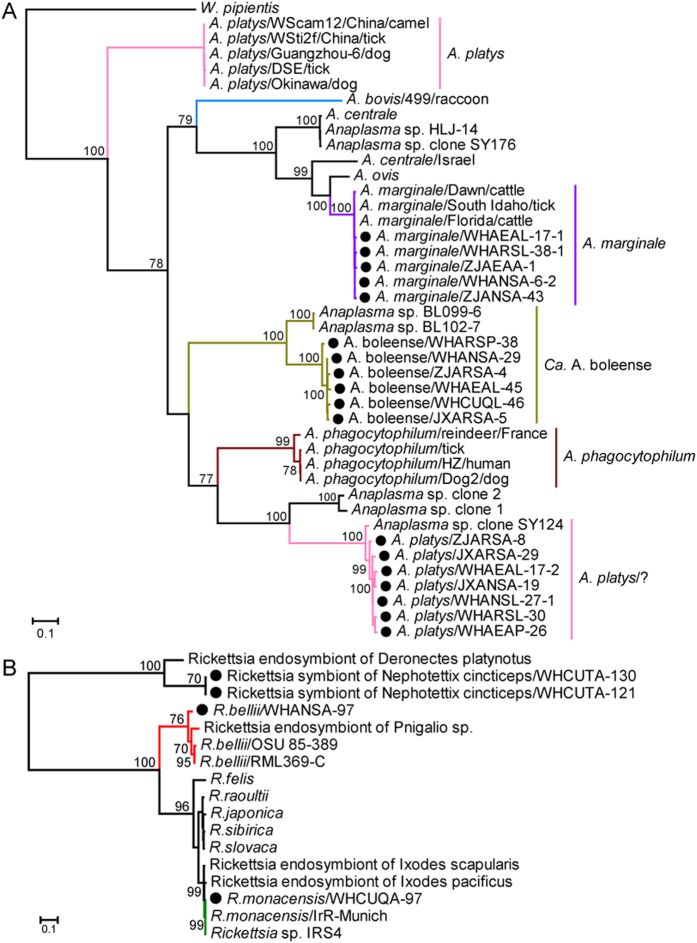
Phylogenetic trees of the citrate synthase gene (*gltA*) of bacteria of the genera *Anaplasma* (**A**) and *Rickettsia* (**B**). The figure description follows that in Fig. 1.

In the *gltA* gene tree, all *Anaplasma* sequences formed three clusters (Fig. 3A). Within the *Candidatus* A. boleense cluster, the bacteria formed a cluster clearly distinct from that previously documented in ticks. Strikingly, although the mosquito *Anaplasma* bacteria (ZJARSA-8, WHANSL-27–1, JXARSA-29, WHAEAL-17-2, JXANSA-19, WHARSL-30, WHAEAP-26) were most closely related to *A. platys* bacteria in both the *rrs* and *groEL* trees, in the *gltA* tree they grouped with strain *Anaplasma* sp. clone SY124 previously identified in ticks from Shenyang of China^36^. Further studies are needed to determine whether these bacteria belong to *A. platys* or represent co-infection (and/or recombinant event). Within the genus *Rickettsia*, the *gltA* gene sequences from mosquitoes were closely related to those from *R. bellii* and *R. monacensis*, respectively, while sequences WHCUTA-121 and WHCUTA-130 formed a distinct lineage (Fig. 3B). As no *gltA* sequences from the *Rickettsia* symbiont of *Nephotettix cincticeps* were available, we could not determine their phylogenetic relationship.

### Rickettsiales in different life stages of mosquitoes sampled in nature

The detection rate of Rickettsiales in adult mosquitoes from our three sampling sites was 19.78% in *An. sinensis*, 23.90% in *Ar. subalbatus*, 3.33% in *Ae. albopictus,* 4.86% in *Cu. quinquefasciatus*, and 6.25% in *Cu. tritaeniorhynchus*. Overall, *Anaplasma* bacteria showed the highest prevalence (10.2%), exhibiting species, geographic and annual variation (Table 1, Supplementary Table S2). In contrast, the detection rates of bacteria from the *Ehrlichia, Candidatus* Neoehrlichia, and *Rickettsia* genera were lower in adult mosquitoes (0.72%, 0.51%, and 1.13%, respectively), possible caused by bias in the PCR assay. Finally, among all the Rickettsiales identified in mosquitoes, *A. marginale* had the highest detection rate (4.43%), followed by *A. phagocytophilum* (2.16%).

To better understand the mosquito circulation of Rickettsiales, eggs, larvae, and pupae were collected from aquatic environments in Wuhan. Although only *A. marginale* and *A. phagocytophilum* were identified in two pools of *An. sinensis* eggs, more Rickettsiales were found in these five mosquito species at the larvae and pupae stages (Table 2, see Supplementary Table S3). In similar pattern to that observed in adult mosquitoes, *Anaplasma* bacteria were at higher prevalence in larvae and pupae than *Ehrlichia, Candidatus* Neoehrlichia, and *Rickettsia*. Notably, *A. phagocytophilum* and *A. marginale* were highly prevalent in larvae. As with the adults, three species of *Ehrlichia (E. chaffeensis, Candidatus* Ehrlichia sp. EHh317, and *Candidatus* Ehrlichia sp. NS101) were detected in the larvae and pupae stages. With the exception of *Ar. subalbatus, Candidatus* N. mikurensis were identified in four other species of mosquitoes. Finally, a novel candidate species (or symbiont/endosybionts) (*Candidatus* Rickettsia sp. Culex tritaeniorhynchus) was identified in *Cu. tritaeniorhynchus* larvae.

In juvenile mosquitoes, the prevalence of these bacteria was high in *Ar. subalbatus* (52.08% larvae and 12.50% in pupae), *An. sinensis* (27.08% in larvae and 4.55% in pupae), and *Ae. albopictus* (18.75% in larvae and 4.86% in pupae), but relatively low in *Cu. quinquefasciatus* (7.64% in larvae and 2.08% in pupae) and *Cu. tritaeniorhynchus* (2.78% in larvae and 2.31% in pupae). In sum, these data suggest that Rickettsiales may be transmitted through the transstadial and/or transovarian transmission routes in mosquitoes, although the number of observations in eggs was small (95% CI 0–24.8%).

More than one species of *Anaplasma* bacteria was detected in some individuals of both adult and juvenile mosquitoes (Table 4), indicative of co-infection. In adult mosquitoes, the most common combinations were *A. marginale* and *A. bovis*, and *A. marginale* with *A. platys*. The co-infection of three bacteria (*A. bovis, A. marginale*, and *A. platys*) was detected in one *An. sinensis* individual. Interestingly, co-infection with *A. phagocytophilum* and *Ehrlichia* sp. EHh317 was also detected in *Ar. subalbatus*. Notably, co-infection was relatively more common in *An. sinensis* mosquitoes, but absent in *Cu. quinquefasciatus* and *Cu. tritaeniorhynchus* mosquitoes. For juvenile mosquitoes, the co-infection of *Anaplasma* bacteria was observed within larvae and pupae of *An. sinensis, Ar. subalbatus* and *Ae. albopictus* mosquitoes, but not in *Cu. quinquefasciatus* and *Cu. tritaeniorhynchus* (Table 4). The most common combination was also *A. marginale* or *A. phagocytophilum* with other bacteria. Finally, co-infection with bacteria from the *Ehrlichia* and *Anaplasma* genera was also detected in juvenile mosquitoes.

### Rickettsiales in different life stages of laboratory reared mosquitoes

To confirm the transstadial and transovarian transmission of Rickettsiales, adult (and/or larvae for *Cu. tritaeniorhynchus*) mosquitoes collected in the field from Wuhan were reared in the laboratory for an entire life cycle (adult-egg-larva-pupae-adult) and tested for the presence of bacteria. From the adult (parent) mosquitoes collected in field, *Ehrlichia* and *Rickettsia* bacteria were identified; *Ehrlichia* sp. EHh317 in *Ae. albopictus, R. japonica* in all five species of mosquitoes, and *R. monacensis* and *R. sibirica* in *Cu. tritaeniorhynchus* (Table 3). Accordingly, these bacteria were also identified in their offspring. Notably, *R. japonica* was detected in all life stages of *Ar. subalbatus* and *Cu. quinquefasciatus*, and in three stages of the other three mosquito species. Additionally, both *R. japonica* and *R. monacensis* were successfully identified at each life stage in *Cu. tritaeniorhynchus*, again supporting the transstadial and transovarial transmission of Rickettsiales. As *Anaplasma* and *Candidatus* N. mikurensis were not identified in adult mosquitos sampled in the field for this experiment, they were similarly not found in laboratory reared eggs, larvae, pupae and adult mosquitoes.

## Discussion

Rickettsiales are associated with a wide range of animals including diverse arthropods, mammals and birds^3^,^6^,^14^,^15^,^16^,^17^,^18^,^19^,^20^. High levels of genetic diversity in Rickettsiales have been identified in both ticks and vertebrates^6^,^14^,^36^, including multiple infection by distinct bacteria in a single tick species^18^,^37^. Our phylogenetic analysis revealed the co-circulation of nine documented and five tentative species bacteria, as well as three symbionts/endosybionts in five species of mosquitoes. Of particular note in this context was that two distinct *Rickettsia* species (or symbionts/endosybionts) were identified in mosquitoes and that a single mosquito can harbor two or more species from the genera *Anaplasma* and *Ehrlichia*. As more than 3,500 species of mosquitoes are distributed worldwide, it is likely that additional (and/or novel) mosquito-associated Rickettsiales (or symbiont/endosybionts) will be discovered in the future.

Ticks are considered the primary vectors for Rickettsiales, especially in the case of *Anaplasma* and *Ehrlichia*^14^,^18^,^19^. Although mosquitoes are both diverse and abundant, with the exception of the genus *Wolbachia*^24^,^26^ there is little evidence for mosquitoes serving as competent vectors or hosts of Rickettsiales bacteria. Previous studies showed that mosquito cell lines (i.e. *Ae. albopictus* and *An. gambiae* cells) could be used to propagate *A. marginal*e, *R. felis, R. montanensis*, and *R. peacockii* bacteria^38^,^39^. Lindh and colleagues also reported the detection of the *rrs* gene of *A. platys and A. ovis* in the *Anopheles* midgut, but could not exclude that the *Anaplasma* bacteria were derived from ingested blood^27^. Recently, Dieme and colleagues reported the transmission potential of *R. felis* infection by *An. gambiae* mosquitoes^28^. Herein, we document the presence of diverse bacteria of the genera *Anaplasma, Ehrlichia, Candidatus* Neoehrlichia and *Rickettsia* in five species of adult and juvenile mosquitoes sampled from three Chinese provinces. In addition, Rickettsiales bacteria were identified in each mosquito life stage (egg, larvae, pupae, and adult). Hence, these data clearly show that diverse Rickettsiales are present in mosquitoes in nature, such that mosquitoes may have played an important role in the transmission, and likely evolution, of Rickettsiales bacteria. In addition, it was noteworthy that some of the Rickettsiales sequences (for example, WHCUTL-65, WHCUTA-121, WHCUTA-130 and WHANSA-146) recovered from mosquitoes were closely related to those of symbionts or endosymbionts. Finally, as only PCR was used here, which may result in some bias, it is possible that the diversity and the prevalence of Rickettsiales bacteria in mosquitoes may be higher than we report.

Intracellular parasites are transmitted by either transstadial and/or transovarial mechanisms (i.e. vertical transmission) in arthropods or by horizontal transmission via infected vertebrates. As horizontal transmission is dependent on the density of susceptible hosts and their intervals of patent infection, the transovarial and/or transstadial pathways may be more reliable routes to transmit intracellular parasites^40^,^41^. Although transovarial and/or transstadial transmission has been documented in members of the *Rickettsiaceae*^15^,^17^,^42^,^43^, this process is not thought to occur to a significant degree in those *Anaplasma* and *Ehrlichia* bacteria documented to date, which may in part be due to lack of the aldolase/adducing domain protein^44^. Hence, the known *Anaplasma* and *Ehrlichia* bacteria (excluding endosymbionts such as Midichloria) transmitted by ticks require feeding on an infected vertebrate^14^. Notably, we identified *Anaplasma, Ehrlichia, Candidatus* Neoehrlichia, and *Rickettsia* bacteria in eggs, larvae, and pupae, as well as adult mosquitoes collected in the field. Additionally, *Ehrlichia* and *Rickettsia* bacteria were identified in eggs, larvae, pupae, and adults reared in the laboratory. Together, these data suggest that Rickettsiales, including *Anaplasma* and *Ehrlichia* spp., may be transmitted transovarially and transstadially in mosquitoes in nature. However, as the detection rates of *Ehrlichia* and *Rickettsia* bacteria in laboratory reared mosquitoes were relatively low, further studies are needed to determine the efficiency of transovarial transmission.

The prevalence of *Anaplasma, Ehrlichia*, and *Rickettsia* bacteria in nature varies substantially with respect to vectors, hosts and geographic regions^4^,^14^,^45^. Similarly, ticks collected at different time points in the same locality can display different infection rates^44^. Such variation can be attributed to several factors, including host susceptibility and competence, the availability of different reservoir hosts, and geo-ecologic factors^6^,^14^,^36^,^45^,^46^,^47^. In addition, adult ticks have an additional blood meal compared to nymphs, such that the infection rates of *A. phagocytophilum* in adult ticks are higher than in nymphs^36^, and because of a lack of transovarial transmission, tick larvae are considered free of *Anaplasmataceae* bacteria^45^. Our analysis of mosquitoes also revealed variation in infection rate according to geographic location, mosquito species and their life stage, and sampling times. For example, infection rates were higher in adult mosquitoes of *An. sinensis* (20.50%) and *Ar. subalbatus* (23.90%), but lower in adult *Ae. Albopictus* (3.33%), *Cu. quinquefasciatus* (4.86%), and *Cu. tritaeniorhynchus* (6.25%), perhaps reflecting further differences in susceptibility. Furthermore, compared with *Ehrlichia, Candidatus* N. mikurensis, and *Rickettsia, Anaplasma* bacteria showed the highest infection rates in adult and juvenile mosquitoes collected in the field.

In all the gene trees inferred here, *A. marginale* from mosquitoes were closely related to strains sampled from cattle^31^. For *Candidatus* A. rodmosense, a close relationship between mosquito- and rodent-associated bacteria is observed in the *rrs* gene tree. Hence, these data are compatible with the inter-species transmission of bacteria among mosquitoes and mammals, such that mosquitoes may act as transmission vectors. However, for other *Anaplasma* bacteria (*Candidatus* A. boleense, *A. bovis,* and *A. platys*), the strains identified here were phylogenetically distinct in all three gene trees, such that additional studies are needed to determine whether they are specifically adapted to mosquitoes.

Bovine anaplasmosis caused by *A. marginale* is widely distributed in tropical and subtropical regions globally, and responsible for substantial economic losses^4^,^48^. Indeed, cattle can develop persistent infections and serve as reservoirs of *A. marginale*^4^. It was therefore notable that the *A. marginale* bacteria identified here were more common than other bacteria in *An. sinensis* and *Ar. subalbatus* mosquitoes, and that these strains are closely related to widely distributed ruminant strains^31^,^49^. Additionally, *An. sinensis* and *Ar. subalbatus* mosquitoes prefer to feed on large animals including cattle, and the geographic distribution of a variety of mosquito species (*An. sinensis, Ar. subalbatus, Ae. albopictus,* and *Cu. quinquefasciatus*) overlaps with the distribution of bovine anaplasmosis in China. Consequently, it is possible that mosquitoes are involved in the spread of bovine anaplasmosis, beyond serving as mechanical fomites.

The identification of extensive genetic diversity of Rickettsiales (especially *Anaplasma*) in adult and juvenile mosquitoes indicates that mosquitoes may have played an important role in the transmission of Rickettsiales bacteria, and that their role as vectors needs to be investigated further. These data are also compatible with the notion that Rickettsiales can be maintained in mosquitoes through transstadial and transovarial transmission. Due to the global distribution of these mosquitoes, greater efforts are clearly needed to determine their role in the evolution and natural transmission of Rickettsiales.

## Material and methods

### Mosquito collection and identification

During the summer (June to August) in 2014 and 2015, 971 adult mosquitoes were collected by ultraviolet light traps from sheep folds, cattle pens, ponds, creeks, and indoors at night in three regions of China: (i) Wuhan city in Hubei province, (ii) Yudu county in Jiangxi province, and (iii) Cixi city in Zhejiang province (see Supplementary Fig. S1). Similarly, mosquito eggs, larvae and pupae were collected from aquatic environments in Wuhan. All mosquitoes were identified to the species level and life stage based on morphologic criteria^50^ and further by molecular differentiation as described previously^51^. The main morphological characters distinguishing mosquito pupae from larvae are that the latter are comma-shaped in their lateral aspect and the head and thorax are merged into a cephalothorax. All samples collected were stored at −80 °C until DNA extraction.

### Mosquito culture

For mosquito culture, adult mosquitoes (fed or unfed, Table 3), including *Ae. albopictus, An. sinensis, Ar. subalbatus* and *Cu. tritaeniorhynchus* collected from the natural aquatic environments in Wuhan were reared for a complete life cycle (adult-egg-larvae-pupae-adult) as described previously^50^,^52^. Larvae and adult *Cu. quinquefasciatus* were also collected for a complete life circle (adult-egg-larvae-pupae-adult or larvae-pupae-adult-egg-larvae-pupae-adult). Samples were collected from adult/larvae mosquitoes caught in the field and at each life stage of cultured mosquitoes.

### DNA extraction, PCR amplification, and sequencing

After washing twice with PBS, adult mosquitoes, larvae and pupae were individually homogenized with a mortar and pestle. Approximately 450 *An. sinensis* and 500 *Cu. tritaeniorhynchus* eggs were pooled (a total of 19 pools) and homogenized. After homogenization, the suspension was incubated at 4 °C for 1 h and centrifuged at 2,500 *g* for 5 min, and the upper fraction collected. DNA was extracted from individual mosquitoes or mosquito pools with the QIAamp DNA Mini Kit (Qiagen GmbH, Hilden, Germany) according to the manufacturer’s instructions, and then subjected to PCR for amplification of both bacterial gene sequences (*rrs, groEL* and *gltA* genes) and mosquito 18S rRNA genes^51^.

Rickettsial DNA was detected using nested PCR targeting a conserved sequence of the Rickettsiales *rrs* gene using the primers Eh-out1/Eh-out2 (outer primers) and Eh-gs1/Eh-gs2 (inner primers)^53^. Primers designed in-house were also used to amplify complete *rrs* gene sequences and partial *groEL* and *gltA* gene sequences by nested PCR. All primer sequemnces are described in Supplementary Table S4.

DNA samples of *E. chaffeensis* strain Arkansas were used as positive controls, with distilled water used as a negative control. Amplified DNA was purified by electrophoresis in low-melting point agarose and ligated into the cloning vector pMD19-T. Subsequently, each vector was transformed into *E. coli* and plated onto agarose culture dishes. Twenty clones were picked from each 20^th^ dish and sent to the Sangon Biotechnology Company (Shanghai, China) for sequencing. To prevent contamination, the pre-PCR mix was prepared in a separate room and template DNA was added using dedicated pipets and tips.

### Sequence data and genetic analyses

DNA sequences of the three bacterial genes obtained here were aligned with existing reference sequences in GenBank using ClustalW (default parameters) as implemented in the MEGA program, version 5.2^54^. Nucleotide and amino acid sequence identities were calculated using DNAStar (DNASTAR, Inc., Madison, WI). Data sets of the following sizes were then used in an evolutionary analysis: (i) a 1425 bp *rrs* alignment (N = 131 sequences); (ii) a 1656 bp *groEL* gene alignment (N = 82); and (iii) a 1227 bp *gltA* gene alignment (N = 59). The sequences recovered in this study were named according to their relatedness to known bacteria, geographic origins, and sample numbers. All sequences obtained here have been submitted to GenBank and assigned accession numbers KU585921-KU586334.

### Phylogenetic analyses

The best-fit evolutionary model for all sequence alignments was determined using jModelTest^55^ and found to be the General Time Reversible (GTR) nucleotide substitution model with a gamma (Γ)-distribution model of among-site rate variation and a proportion of invariable sites (i.e. GTR + Γ + I). Phylogenetic trees using this model were then estimated using the Maximum Likelihood (ML) method implemented in PhyML (version 3)^56^. All trees were mid-point rooted for clarity only.

## Additional Information

**How to cite this article**: Guo, W.-P. *et al*. Extensive genetic diversity of Rickettsiales bacteria in multiple mosquito species. *Sci. Rep.* **6**, 38770; doi: 10.1038/srep38770 (2016).

**Publisher's note:** Springer Nature remains neutral with regard to jurisdictional claims in published maps and institutional affiliations.

## Supplementary Material

Supplementary Information

## Figures and Tables

**Table 1:** Prevalence of Rickettsiales bacteria in adult mosquitoes collected in Hubei, Jiangxi, and Zhejiang provinces, China, during 2014–2015.

Species	*Location*	*Anaplasma*^a^						*Ehrlichia*			*Rickettsia*				*C.N.mik*	Total^b^
		*C.A.bol*	*A.bov*	*A.mar*	*A.pha*	*A.pla*	*C.A.rod*	*E.cha*	*E.ehh*	*E.ns*	*C.R.as*	*R.bel*	*R.mon*	*R.nc*		
Ae.a.	Hubei	0	0	0	0	0	0	0	0	0	0	0	0	0	1	1/144
	Zhejiang	1	0	2	1	0	0	0	0	0	0	0	0	0	0	4/6
An.s.	Hubei	1	5	25	0	8	2	0	1	0	1	1	0	0	2	46/192
	Jiangxi	0	2	4	0	2	0	0	0	0	0	0	0	0	0	8/38
	Zhejiang	0	0	1	0	0	0	0	0	0	0	0	0	0	0	1/48
Ar.s.	Hubei	0	5	4	4	3	1	0	2	1	0	0	0	0	7	27/103
	Jiangxi	2	0	0	4	1	0	0	0	0	0	0	0	0	0	7/42
	Zhejiang	1	0	0	1	2	0	0	0	0	0	0	0	0	0	4/14
Cu.q.	Hubei	0	0	0	0	0	2	1	2	0	0	0	1	0	1	7/144
Cu.t.	Hubei	0	0	2	1	5	0	0	0	0	0	0	0	2	0	10/144
	Zhejiang	0	0	5	0	0	0	0	0	0	0	0	0	0	0	5/96
Total		5	12	43	11	21	5	1	5	1	1	1	1	2	11	120/971

^a^Mosquito species are abbreviated as follows: *Aedes albopictus*, Ae.a.; *Anopheles sinensis*, An.s.; *Armigeres subalbatus*, Ar.s.; *Culex quinquefasciatus*, Cu.q.; *Culex tritaeniorhynchus*, Cu.t. Bacterial species are abbreviated as follows: *Candidatus* Anaplasma boleense, *C.* A.bol; *Anaplasma bovis, A.bov; Anaplasma marginale, A.mar; Anaplasma phagocytophilum, A.pha; Anaplasma platys, A.pla; Candidatus* Anaplasma rodmosense, *C.*A.rod; *Ehrlichia chaffensis, E.cha; Ehrlichia* sp. EHh317, E.ehh; *Ehrlichia* sp. NS101, E.ns; *Candidatus* Rickettsia sp. Anopheles sinensis, *C*. R.as; *Rickettsia bellii, R.bel; Rickettsia monacensis, R.mon; Rickettsia* symbiont of Nephotettix cincticeps, R.nc; *Candidatus* Neoehrlichia mikurensis, *C.*N.mik.

^b^PCR positive/mosquitoes collected.

**Table 2 t2:** Prevalence of Rickettsiales bacteria in eggs, larvae and pupae collected from aquatic environments in Wuhan (Hubei province), China, during 2014–2015.

Species	*Anaplasma*^a^						*Ehrlichia*			*Rickettsia* *C.*R.ct	C.N.mik	Total (mean ± 95% CI)
	*C.A.bol*	*A.bov*	*A.mar*	*A.pha*	*A.pla*	*C.*A.rod	*E.cha*	E.ehh	E.ns			
Egg												
*Ae.a*	0	0	0	0	0	0	0	0	0	0	0	0/0
* An.s*	0	0	1	1	0	0	0	0	0	0	0	2/9 (10.5 ± 13.8)
* Ar.s*	0	0	0	0	0	0	0	0	0	0	0	0/0
* Cu.q*	0	0	0	0	0	0	0	0	0	0	0	0/0
* Cu.t*	0	0	0	0	0	0	0	0	0	0	0	0/10
* *Subtotal	0	0	1	1	0	0	0	0	0	0	0	2/19 (10.5 ± 13.8)
Larvae												
* Ae.a*	1	4	4	7	1	1	0	6	1	0	2	27/144 (18.8 ± 6.5)
* An.s*	0	2	5	5	1	0	0	0	0	0	0	13/48 (27.1 ± 12.6)
* Ar.s*	1	6	6	10	2	0	0	0	0	0	0	25/48 (52.1 ± 14.4)
* Cu.q*	1	0	2	3	0	2	1	0	0	0	2	11/144 (7.6 ± 4.4)
* Cu.t*	0	0	0	0	2	0	0	0	0	1	1	4/144 (2.8 ± 2.7)
* *Subtotal	3	12	17	25	6	3	1	6	1	1	5	80/528 (15.1 ± 3.1)
Pupae												
* Ae.a*	0	0	1	0	1	1	0	2	0	0	2	7/144 (2.8 ± 2.7)
* An.s*	1	0	1	0	1	0	0	0	0	0	1	4/88 (4.5 ± 4.4)
* Ar.s*	1	2	2	0	1	0	0	0	0	0	0	6/48 (12.5 ± 9.5)
* Cu.q*	0	1	0	0	0	0	0	1	0	0	1	3/144 (2.1 ± 2.4)
* Cu.t*	0	0	0	0	0	0	1	0	0	0	2	3/130 (2.3 ± 2.6)
* *Subtotal	2	3	4	0	3	1	1	3	0	0	6	23/554 (4.2 ± 1.7)

^a^Abbreviations are the same as those given in Table 1. *Candidatus* Rickettsia sp. Culex tritaeniorhynchus, *C*. R.ct.

**Table 3 t3:** Co-circulation of Rickettsiales in eggs, larvae, pupae, and adult mosquitoes reared in the laboratory.

Species			*Ehrlichia*	*Rickettsia*			Total
	Life stage		*Ehrlichia* sp. EHh317	*R. japonica*	*R. monacensis*	*R. sibirica*	
*Ae. albopictus*	Parent	Adult	1	8	0	0	9/84
	Offspring	Egg	0	0	0	0	0/4
		Larvae	2	10	0	0	12/57
		Pupae	0	2	0	0	2/105
		Adult	0	1	0	0	1/66
*An. sinensis*	Parent	Adult	0	4	0	0	4/120
	Offspring	Egg	0	0	0	0	0/7
		Larvae	0	3	0	0	3/120
		Pupae	0	4	0	0	4/114
		Adult	0	1	0	0	1/120
*Ar. subalbatus*	Parent	Adult	0	4	0	0	4/120
	Offspring	Egg	0	1	0	0	1/7
		Larvae	0	8	0	0	8/146
		Pupae	0	7	0	0	7/116
		Adult	0	1	0	0	1/118
*Cu. quinquefasciatus*	Parent	Larvae	0	5	0	0	5/120
		Adult	0	6	0	0	6/120
	Offspring	Egg	0	2	0	0	2/19
		Larvae	0	3	0	0	3/120
		Pupae	0	6	0	0	6/106
		Adult	0	1	0	0	1/138
*Cu. tritaeniorhynchus*	Parent	Adult	0	3	1	1	5/216
	Offspring	Egg	0	0	1	1	2/5
		Larvae	0	2	2	2	6/120
		Pupae	0	1	10	1	12/120
		Adult	0	15	1	6	22/120
Total			3	98	15	11	127/2508

**Table 4:** Co-infection of Rickettsiales in adult, larve and pupae mosquitoes collected in Hubei, Jiangxi, and Zhejiang provinces, China, during 2014–2015.

Species	Location	Life stage	Bacteria	PCR positive/Mosquitoes collected (%)
*Ae. albopictus*	Zhejiang	Adult	*C*. A. boleense, *A. marginale*	1/6 (16.67)
	Hubei	Larvae	*A. marginale, A. phagocytophilum*	1/144 (0.69)
			*A. bovis, A. marginale*	1/144 (0.69)
			*A. marginale, Ehrlichia* sp. EHh317	1/144 (0.69)
			*A. bovis, A. phagocytophilum, Ehrlichia* sp. EHh317	1/144 (0.69)
*An. sinensis*	Hubei	Adult	*C*. A. boleense, *A. marginale*	1/192 (0.52)
			*A. bovis, A. marginale*	3/192 (1.56)
			*A. bovis, A. platys*	1/192 (0.52)
			*A. marginale, A. platys*	3/192 (1.56)
			*A. bovis, A. marginale, A. platys*	1/192 (0.52)
		Larvae	*A. bovis, A. marginale*	2/48 (4.2)
			*A. marginale, A. phagocytophilum*	1/48 (2.1)
			*A. marginale, A. platys*	1/48 (2.1)
		Pupae	*C*. A. boleense, *A. phagocytophilum*	1/88 (1.0)
	Jiangxi		*A. bovis, A. platys*	2/38 (5.26)
*Ar. subalbatus*	Hubei	Adult	*A. bovis, A. marginale*	2/103 (1.94)
			*A. marginale, A. platys*	2/103 (1.94)
			*A. phagocytophilum, Ehrlichia* sp. EHh317	1/103 (0.97)
		Larvae	*A. marginale, A. phagocytophilum*	3/48 (6.25)
			*C*. A. boleense, *A. phagocytophilum*	1/48 (2.08)
			*A. bovis, A. phagocytophilum*	1/48 (2.08)
			*A. bovis, A. marginale*	2/48 (4.17)
		Pupae	*A. bovis, A. marginale*	1/48 (2.08)
