# Supplementary material for: Extensive genetic diversity of Rickettsiales bacteria in multiple mosquito species
Source: Sci Rep. 2016 Dec 9;6:38770. doi: 10.1038/srep38770 (PMC5146937; doi:10.1038/srep38770)
Supplement: Supplementary Information [file srep38770-s1.pdf]

Extensive genetic diversity of Rickettsiales bacteria in multiple mosquito species

Wen-Ping Guo[a1], Jun-Hua Tian[b1], Xian-Dan Lin[c], Xue-Bing Ni[a], Xiao-Ping Chen[a], Yong Liao[d], Si-Yuan Yang[a], J. Stephen Dumler[e], Edward C. Holmes[af], Yong-Zhen Zhang[a2]

[a]State Key Laboratory of Infectious Disease Prevention and Control, Department of Zoonoses, National Institute for Communicable Disease Control and Prevention, Chinese Center for Disease Control and Prevention, Beijing; Collaborative Innovation Center for Diagnosis and Treatment of Infectious Diseases, Hangzhou, China.

[b]Wuhan Center for Disease Control and Prevention, Wuhan, Hubei Province, China.

[c]Wenzhou Center for Disease Control and Prevention, Wenzhou, Zhejiang Province, China.

[d]Ganzhou Center for Disease Control and Prevention, Ganzhou, Jiangxi Province, China.

[e]Department of Pathology, Uniformed Services University for the Health Sciences, Bethesda, MD 20814, USA.

[f]Marie Bashir Institute for Infectious Diseases and Biosecurity, Charles Perkins Centre, School of Life and Environmental Sciences and Sydney Medical School, The University of Sydney, Sydney, NSW 2006, Australia.

[1]These authors contributed equally to this article.

[2]Correspondence to: Dr. Yong-Zhen Zhang, State Key Laboratory of Infectious Disease Prevention and Control, National Institute of Communicable Disease Control and Prevention, Chinese Center for Disease Control and Prevention, Changping Liuzi 5, Beijing, 102206, China. Tel: 086-10-58900782; Email: zhangyongzhen@icdc.cn.

Supplementary Table S1. Bacterial sequences obtained in this study.

**Strains**	**Genes(nt)**			**Bacteria**[a]
	rrs	*gltA*	*groEL*	
Anopheles sinensis				
Egg				
WHANSE-2	1400 (99.2%)[b]	-[c]	-	*A. platys*
WHANSE-2-1	330 (99.1%)	-	-	*A. marginale*
Larvae				
WHANSL-11-1	343 (100%)	-	-	*A. marginale*
WHANSL-11	345 (97.0%)	-	-	*A. phagocytophilum*
WHANSL-24-1	342 (98.5%)	-	-	*A. marginale*
WHANSL-24	345 (98.6%)	-	-	*A. bovis*
WHANSL-27-1	1430 (99.6%)	838 (58.5%)	1050 (84.9%)	*A. platys*
WHANSL-27-2	1380 (99.5%)	-	862 (98.8%)	*A. marginale*
WHANSL-30	337 (98.8%)	-	-	*A. phagocytophilum*
WHANSL-34	346 (96.4%)	-	-	*A. phagocytophilum*
WHANSL-36	353 (98.0%)	-	-	*A. phagocytophilum*
WHANSL-41	343 (99.1%)	-	-	*A. marginale*
WHANSL-8-1	342 (99.7%)	-	-	*A. marginale*
WHANSL-8	1440 (97.8%)	-	-	*A. bovis*
WHANSL-16	355 (96.1%)	-	-	*A. phagocytophilum*
WHANSL-LabF50	650 (100%)	-	-	*R. japonica*
WHANSL-LabF52	650 (100%)	-	-	*R. japonica*
WHANSL-LabF59	650 (100%)	-	-	*R. japonica*
Pupae				
WHANSP-48-1	342 (96.8%)	-	-	*A. phagocytophilum*
WHANSP-48	1402 (99.4%)	-	580 (92.7%)	*C.* A. boleense
WHANSP-5-1	342 (99.1%)	-	-	*A. marginale*
WHANSP-60	240 (98.7%)	-	-	*C.* N. mik
WHANSP-LabF85	1100 (99.6%)	-	-	*R. japonica*
WHANSP-LabF100	650 (100%)	-	-	*R. japonica*
WHANSP-LabF101	1100 (100%)	-	-	*R. japonica*
WHANSP-LabF106	650 (100%)	-	-	*R. japonica*
Adult				
JXANSA-19-1	400 (99.2)	-	-	*A. bovis*
JXANSA-19	1434 (99.3%)	832 (57.6%)	1035 (83.6%)	*A. platys*
JXANSA-2	1378 (99.3%)	-	859 (98.7%)	*A. marginale*
JXANSA-24	1377 (99.1%)	-	-	*A. marginale*
JXANSA-30-1	381 (98.4%)	-	-	*A. bovis*
JXANSA-30	340 (98.8%)	-	-	*A. platys*
JXANSA-34	333 (99.4%)	-	-	*A. marginale*
JXANSA-8	333 (100%)	-	-	*A. marginale*

(*Continued*)

**Strains**	**Genes(nt)**			**Bacteria**
	rrs	*gltA*	*groEL*	
ZJANSA-43	1442 (99.6%)	1186 (99.6%)	862 (98.8%)	*A. marginale*
WHANSA-12	978 (99.6%)	-	-	*A. marginale*
WHANSA-13	1428 (99.5%)	-	-	*A. marginale*
WHANSA-15	1439 (99.9%)	-	-	*A. marginale*
WHANSA-19	898 (99.8%)	-	-	*A. marginale*
WHANSA-22	953 (99.6%)	-	-	*A. marginale*
WHANSA-24-1	1348 (98.5%)	-	625 (86.4%)	*A. bovis*
WHANSA-24-2	1438 (99.2%)	-	1020 (84.9%)	*A. platys*
WHANSA-29	1402 (99.7%)	685 (83.2%)	491 (91.0%)	*C.* A. boleense
WHANSA-29-12	1381 (99.8%)	-	-	*A. marginale*
WHANSA-36	1349 (99.9%)	-	-	*A. marginale*
WHANSA-37	1361 (99.9%)	-	-	*A. marginale*
WHANSA-38	1429 (99.9%)	-	-	*A. marginale*
WHANSA-39	1432 (99.4%)	-	-	*A. marginale*
WHANSA-40	1433 (99.2%)	-	-	*A. platys*
WHANSA-45-1	1393 (98.1%)	-	-	*A. platys*
WHANSA-45-2	1440 (98.5%)	-	-	*A. marginale*
WHANSA-47-1	990 (99.8%)	-	-	*A. marginale*
WHANSA-48	1409 (99.6%)	-	-	*A. marginale*
WHANSA-52-1	466 (99.8%)	-	-	*A. marginale*
WHANSA-52	454 (98.4%)	-	-	*A. bovis*
WHANSA-53-2	526 (98.7%)	-	-	*A. marginale*
WHANSA-53	525 (99.2%)	-	-	*A. bovis*
WHANSA-6-1	1436 (98.6%)	-	628 (87.2%)	*A. bovis*
WHANSA-6-2	1442 (99.7%)	1219 (99.6%)	-	*A. marginale*
WHANSA-6-3	1437 (99.2%)	-	1050 (84.7%)	*A. platys*
WHANSA-60-2	595 (99.5%)	-	-	*A. marginale*
WHANSA-63-1	582 (100%)	-	-	*A. marginale*
WHANSA-63	519 (99.4%)	-	-	*A. platys*
WHANSA-7-4	1427 (97.9%)	-	-	*A. platys*
WHANSA-71-1	516 (99.8%)	-	-	*A. marginale*
WHANSA-71	555 (99.5%)	-	-	*A. platys*
WHANSA-79	509 (98.6%)	-	-	*A. marginale*
WHANSA-8-2	1429 (99.5%)	-	-	*A. marginale*
WHANSA-8	1430 (99.4%)	-	1020 (84.4%)	*A. platys*
WHANSA-82-1	573 (98.6%)	-	-	*A. bovis*
WHANSA-82	658 (99.2%)	-	-	*A. marginale*
WHANSA-83	398 (99.7%)	-	-	*A. marginale*
WHANSA-89	395 (99.0%)	-	-	*A. marginale*

(*Continued*)

**Strains**	**Genes(nt)**			**Bacteria**
	rrs	*gltA*	*groEL*	
WHANSA-92	381 (99.2%)	-	-	*A. marginale*
WHANSA-122	240 (100%)	-	-	*C.* A. rodmosense
WHANSA-161	240 (100%)	-	-	*C.* A. rodmosense
Ehrlichia sp. WHANSA-181	650 (99.8%)	-	-	*Ehrlichia* sp. EHh317
WHANSA-97	1300 (99.9%)	1000 (97.4%)	400 (96.6%)	*R. bellii*
WHANSA-146	1300 (98.4%)	-	-	*C.* R. as
WHANSA-108	240 (98.7%)	-	-	*C.* N. mik
WHANSA-133	240 (98.7%)	-	-	*C.* N. mik
WHANSA-LabF24	650 (100%)	-	-	*R. japonica*
WHANSA-LabP60	650 (99.5%)	-	-	*R. japonica*
WHANSA-LabP66	650 (100%)	-	-	*R. japonica*
WHANSA-LabP86	650 (100%)	-	-	*R. japonica*
WHANSA-LabP95	650 (100%)	-	-	*R. japonica*
Armigeres subalbatus				
Egg				
WHARSE-LabF5	650 (100%)	-	-	*R. japonica*
Larvae				
WHARSL-11-1	336 (97.6%)	-	-	*A. phagocytophilum*
WHARSL-11	343 (97.4%)	-	-	*A. bovis*
WHARSL-12	342 (97.7%)	-	-	*A. phagocytophilum*
WHARSL-16-1	374 (98.7%)	-	-	*A. phagocytophilum*
WHARSL-18-1	361 (99.4%)	-	-	*A. marginale*
WHARSL-18	340 (97.4%)	-	-	*A. phagocytophilum*
WHARSL-2-1	389 (99.2%)	-	-	*A. phagocytophilum*
WHARSL-2	1342 (99.3%)	-	563 (92.0%)	*C.* A. boleense
WHARSL-21-1	373 (99.2%)	-	-	*A. marginale*
WHARSL-22	342 (97.4%)	-	-	*A. bovis*
WHARSL-24	343 (98.0%)	-	-	*A. bovis*
WHARSL-26-1	351 (99.4%)	-	-	*A. marginale*
WHARSL-26	336 (97.3%)	-	-	*A. phagocytophilum*
WHARSL-28-1	372 (98.4%)	-	-	*A. bovis*
WHARSL-28	970 (99.6%)	-	-	*A. marginale*
WHARSL-3-1	375 (99.7%)	-	-	*A. marginale*
WHARSL-3	324 (97.8%)	-	-	*A. phagocytophilum*
WHARSL-30	1381 (99.3%)	826 (57.6%)	1041 (84.9%)	*A. platys*
WHARSL-32	333 (98.8%)	-	-	*A. platys*
WHARSL-33	343 (97.1%)	-	-	*A. bovis*
WHARSL-34	343 (96.8%)	-	-	*A. phagocytophilum*
WHARSL-35	332 (97.6%)	-	-	*A. phagocytophilum*

(*Continued*)

**Strains**	**Genes(nt)**			**Bacteria**
	rrs	*gltA*	*groEL*	
WHARSL-38-1	1376 (99.8%)	1180 (99.5%)	850 (98.6%)	*A. marginale*
WHARSL-38-2	1441 (98.5%)	-	628 (86.9%)	*A. bovis*
WHARSL-9	335 (97.9%)	-	-	*A. phagocytophilum*
WHARSL-LabF8	650 (100%)	-	-	*R. japonica*
WHARSL-LabF12	650 (100%)	-	-	*R. japonica*
WHARSL-LabF14	650 (100%)	-	-	*R. japonica*
WHARSL-LabF21	650 (100%)	-	-	*R. japonica*
WHARSL-LabF34	650 (100%)	-	-	*R. japonica*
WHARSL-LabF56	650 (100%)	-	-	*R. japonica*
WHARSL-LabF115	650 (100%)	-	-	*R. japonica*
WHARSL-LabF130	650 (100%)	-	-	*R. japonica*
Pupae				
WHARSP-17	1381 (99.4%)	-	993 (85.1%)	*A. platys*
WHARSP-19	1378 (99.9%)	-	850 (98.6%)	*A. marginale*
WHARSP-2-1	371 (98.9%)	-	-	*A. bovis*
WHARSP-30-1	372 (98.7%)	-	-	*A. bovis*
WHARSP-30-2	343 (98.8%)	-	-	*A. marginale*
WHARSP-38	1372 (99.6%)	954 (84.2%)	-	*C.* A. boleense
WHARSP-LabF3	650 (100%)	-	-	*R. japonica*
WHARSP-LabF11	650 (100%)	-	-	*R. japonica*
WHARSP-LabF18	650 (100%)	-	-	*R. japonica*
WHARSP-LabF21	650 (100%)	-	-	*R. japonica*
WHARSP-LabF23	650 (100%)	-	-	*R. japonica*
WHARSP-LabF39	650 (100%)	-	-	*R. japonica*
WHARSP-LabF42	650 (100%)	-	-	*R. japonica*
Adult				
JXARSA-1	348 (99.4%)	-	-	*A. phagocytophilum*
JXARSA-15	354 (99.2%)	-	-	*A. phagocytophilum*
JXARSA-2	356 (99.1%)	-	-	*A. phagocytophilum*
JXARSA-29	1359 (99.2%)	832 (58.2%)	866 (86.7%)	*A. platys*
JXARSA-32	343 (99.4%)	-	-	*A. phagocytophilum*
JXARSA-33	1360 (99.3%)	679 (83.2%)	530 (92.6%)	*C.* A. boleense
JXARSA-5	1371 (99.1%)	678 (83.2%)	-	*C.* A. boleense
WHARSA-14-1	321 (99.4%)	-	-	*A. marginale*
WHARSA-14	1002 (98.8%)	-	1017 (84.2%)	*A. platys*
WHARSA-30-1	361 (98.9%)	-	-	*A. bovis*
WHARSA-30	1380 (99.9%)	-	850 (98.0%)	*A. marginale*
WHARSA-40-1	1380 (99.7%)	-	835 (98.6%)	*A. marginale*
WHARSA-40-2	1349 (98.5%)	-	628 (86.3%)	*A. bovis*

(*Continued*)

**Strains**	**Genes(nt)**			**Bacteria**
	rrs	*gltA*	*groEL*	
WHARSA-47-1	1380 (99.5%)	-	-	*A. platys*
WHARSA-47-2	1378 (99.9%)	-	-	*A. marginale*
WHARSA-5	366 (98.9%)	-	-	*A. phagocytophilum*
WHARSA-7	1380 (99.6%)	-	1047 (83.7%)	*A. platys*
ZJARSA-11	358 (98.9%)	-	-	*A. platys*
ZJARSA-2	349 (98.6%)	-	-	*A. phagocytophilum*
ZJARSA-4	1393 (99.3%)	685 (82.5%)	491 (91.0%)	*C.* A. boleense
ZJARSA-8	1380 (99.6)	618 (56.5%)	987 (84.6%)	*A. platys*
WHARSA-80	240 (100%)	-	-	*C.* A. rodmosense
WHARSA-86	650 (99.1%)	-	-	*A. phagocytophilum*
WHARSA-103	650 (99.2%)	-	-	*A. phagocytophilum*
WHARSA-106	650 (98.9%)	-	-	*A .bovis*
WHARSA-121	650 (99.2%)	-	-	*A .bovis*
WHARSA-132	650 (99.2%)	-	-	*A .bovis*
WHARSA-87-1	650 (99.3%)	-	-	*A. phagocytophilum*
Ehrlichia sp. WHARSA-87	650 (99.6%)	-	450 (89.4%)	*Ehrlichia* sp. EHh317
Ehrlichia sp. WHARSA-128	650 (99.4%)	-	-	*Ehrlichia* sp. NS101
Ehrlichia sp. WHARSA-134	1110 (99.7%)	-	-	*Ehrlichia* sp. EHh317
WHARSA-85	1110 (99.3%)	-	-	*C.* N. mik
WHARSA-107	650 (99.6%)	-	-	*C.* N. mik
WHARSA-115	650 (99.6%)	-	-	*C.* N. mik
WHARSA-118	650 (99.6%)	-	-	*C.* N. mik
WHARSA-135	650 (99.6%)	-	-	*C.* N. mik
WHARSA-140	650 (99.6%)	-	-	*C.* N. mik
WHARSA-142	650 (99.6%)	-	-	*C.* N. mik
WHARSA-LabF12	650 (100%)	-	-	*R. japonica*
WHARSA-LabP24	650 (100%)	-	-	*R. japonica*
WHARSA-LabP51	650 (100%)	-	-	*R. japonica*
WHARSA-LabP70	650 (100%)	-	-	*R. japonica*
WHARSA-LabP71	650 (100%)	-	-	*R. japonica*
Aedes albopictus				
Larvae				
WHAEAL-10	357 (99.2%)	-	-	*A. phagocytophilum*
WHAEAL-14	346 (99.3%)	-	-	*A. phagocytophilum*
WHAEAL-16	357 (99.3%)	-	-	*A. phagocytophilum*
WHAEAL-17-1	1346 (99.3%)	1201 (99.5%)	856 (98.6%)	*A. marginale*
WHAEAL-17-2	938 (99.2%)	832 (57.9%)	1014 (84.5%)	*A. platys*
WHAEAL-23	356 (99.2%)	-	-	*A. phagocytophilum*
WHAEAL-25	339 (99.4%)	-	-	*A. phagocytophilum*

(*Continued*)

**Strains**	**Genes(nt)**			**Bacteria**
	rrs	*gltA*	*groEL*	
WHAEAL-45	1356 (99.2%)	685 (82.5%)	-	*C.* A. boleense
WHAEAL-7-1	345 (99.4%)	-	-	*A. bovis*
WHAEAL-7-2	374 (99.2%)	-	-	*A. marginale*
WHAEAL-69	650 (98.8%)	-	-	*A. phagocytophilum*
WHAEAL-81	650 (99.8%)	-	-	*A. marginale*
WHAEAL-84	550 (99.1%)	-	-	*A. bovis*
WHAEAL-109	650 (99.1%)	-	-	*A .bovis*
WHAEAL-112	240 (100%)	-	-	*C.* A. rodmosense
WHAEAL-56	535 (99.6%)	-	-	*A. marginale*
WHAEAL-79-1	668 (99.6%)	-	-	*A. phagocytophilum*
WHAEAL-79-2	667 (98.9%)	-	-	*A. bovis*
Ehrlichia sp. WHAEAL-56-1	650 (99.9%)	-	-	*Ehrlichia* sp. EHh317
Ehrlichia sp. WHAEAL-79	650 (100%)	-	450 (89.4%)	*Ehrlichia* sp. EHh317
Ehrlichia sp. WHAEAL-110	650 (99.9%)	-	-	*Ehrlichia* sp. EHh317
Ehrlichia sp. WHAEAL-113	650 (99.2%)	-	-	*Ehrlichia* sp. NS101
Ehrlichia sp. WHAEAL-132	650 (99.1%)	-	450 (89.2%)	*Ehrlichia* sp. EHh317
Ehrlichia sp. WHAEAL-139	1110 (99.2%)	-	-	*Ehrlichia* sp. EHh317
Ehrlichia sp. WHAEAL-144	1110 (99.3%)	-	-	*Ehrlichia* sp. EHh317
WHAEAL-125	1110 (98.8%)	-	-	*C.* N. mik
WHAEAL-130	650 (99.6%)	-	-	*C.* N. mik
WHAEAL-LabF6	650 (100%)	-	-	*R. japonica*
WHAEAL-LabF18	650 (100%)	-	-	*R. japonica*
WHAEAL-LabF21	650 (100%)	-	-	*R. japonica*
WHAEAL-LabF34	650 (100%)	-	-	*R. japonica*
WHAEAL-LabF37	650 (100%)	-	-	*R. japonica*
WHAEAL-LabF39	1100 (100%)	-	-	*R. japonica*
WHAEAL-LabF49	650 (100%)	-	-	*R. japonica*
WHAEAL-LabF50	650 (100%)	-	-	*R. japonica*
WHAEAL-LabF52	650 (100%)	-	-	*R. japonica*
WHAEAL-LabF54	650 (100%)	-	-	*R. japonica*
Ehrlichia sp. WHAEAL-LabF19	650 (99.4%)	-	-	*Ehrlichia* sp. EHh317
Ehrlichia sp. WHAEAL-LabF20	650 (99.4%)	-	-	*Ehrlichia* sp. EHh317
Pupae				
WHAEAP-26	938 (98.9%)	823 (57.7%)	1047 (83.5%)	*A. platys*
WHAEAP-33	1380 (99.7%)	-	850 (98.4%)	*A. marginale*
WHAEAP-67	240 (100%)	-	-	*C.* A. rodmosense
Ehrlichia sp. WHAEAP-65	240 (100%)	-	-	*Ehrlichia* sp. EHh317
Ehrlichia sp. WHAEAP-69	240 (100%)	-	-	*Ehrlichia* sp. EHh317
WHAEAP-55	240 (98.7%)	-	-	*C.* N. mik

(*Continued*)

**Strains**	**Genes(nt)**			**Bacteria**
	rrs	*gltA*	*groEL*	
WHAEAP-61	240 (98.7%)	-	-	*C.* N. mik
WHAEAP-LabF43	650 (100%)	-	-	*R. japonica*
WHAEAP-LabF45	650 (100%)	-	-	*R. japonica*
Adult				
ZJAEAA-1	1382 (99.7%)	1195 (99.5%)	817 (98.5%)	*A. marginale*
ZJAEAA-4	345 (99.4%)	-	-	*A. phagocytophilum*
ZJAEAA-5-1	377 (99.5%)	-	-	*A. marginale*
ZJAEAA-5	1379 (99.4%)	666 (84.5%)	491 (92.0%)	*C.* A. boleense
WHAEAA-39	240 (98.7%)	-	-	*C.* N. mik
WHAEAA-LabF26	1100 (99.8%)	-	-	*R. japonica*
WHAEAA-LabP5	650 (100%)	-	-	*R. japonica*
WHAEAA-LabP12	650 (100%)	-	-	*R. japonica*
Ehrlichia sp. WHAEAA-LabP15	650 (99.6%)	-	-	*Ehrlichia* sp. EHh317
WHAEAA-LabP33	650 (100%)	-	-	*R. japonica*
WHAEAA-LabP44	650 (99.5%)	-	-	*R. japonica*
WHAEAA-LabP57	650 (99.5%)	-	-	*R. japonica*
WHAEAA-LabP58	650 (99.5%)	-	-	*R. japonica*
WHAEAA-LabP68	650 (100%)	-	-	*R. japonica*
WHAEAA-LabP82	650 (99.5%)	-	-	*R. japonica*
Culex quinquefasciatus				
Egg				
WHCUQE-LabF3	650 (100%)	-	-	*R. japonica*
WHCUQE-LabF19	650 (100%)	-	-	*R. japonica*
Larvae				
WHCUQL-18	356 (97.2%)	-	-	*A. phagocytophilum*
WHCUQL-2	356 (98.9%)	-	-	*A. phagocytophilum*
WHCUQL-4	356 (98.0%)	-	-	*A. phagocytophilum*
WHCUQL-46	1323 (99.4%)	682 (82.5%)	-	*C.* A. boleense
WHCUQL-65	240 (100%)	-	-	*C.* A. rodmosense
WHCUQL-98	240 (100%)	-	-	*A. marginale*
WHCUQL-112	240 (97.3%)	-	-	*C.* A. rodmosense
WHCUQL-118	240 (98.3%)	-	-	*A. marginale*
WHCUQL-83	240 (100%)	-	-	*E. chaffeensis*
WHCUQL-53	240 (98.7%)	-	-	*C.* N. mik
WHCUQL-110	240 (98.7%)	-	-	*C.* N. mik
WHCUQL-LabF8	650 (100%)	-	-	*R. japonica*
WHCUQL-LabF14	650 (100%)	-	-	*R. japonica*
WHCUQL-LabF89	650 (100%)	-	-	*R. japonica*
WHCUQL-LabP6	650 (100%)	-	-	*R. japonica*

(*Continued*)

**Strains**	**Genes(nt)**			**Bacteria**
	rrs	*gltA*	*groEL*	
WHCUQL-LabP11	650 (99.5%)	-	-	*R. japonica*
WHCUQL-LabP27	650 (100%)	-	-	*R. japonica*
WHCUQL-LabP34	650 (99.5%)	-	-	*R. japonica*
WHCUQL-LabP39	650 (100%)	-	-	*R. japonica*
WHCUQL-LabP60	650 (100%)	-	-	*R. japonica*
Pupae				
WHCUQP-88	650 (99.2%)	-	-	*A. bovis*
Ehrlichia sp. WHCUQP-70	240 (100%)	-	-	*Ehrlichia* sp. EHh317
WHCUQP-78	240 (98.7%)	-	-	*C.* N. mik
WHCUQP-LabF5	650 (100%)	-	-	*R. japonica*
WHCUQP-LabF7	650 (100%)	-	-	*R. japonica*
WHCUQP-LabF68	1100 (99.5%)	-	-	*R. japonica*
WHCUQP-LabF78	650 (100%)	-	-	*R. japonica*
WHCUQP-LabF79	650 (100%)	-	-	*R. japonica*
WHCUQP-LabF81	650 (100%)	-	-	*R. japonica*
Adult				
WHCUQA-73	240 (100%)	-	-	*C.* A. rodmosense
WHCUQA-81	240 (100%)	-	-	*C.* A. rodmosense
WHCUQA-68	240 (100%)	-	-	*E. chaffeensis*
Ehrlichia sp. WHCUQA-72	240 (100%)	-	-	*Ehrlichia* sp. EHh317
Ehrlichia sp. WHCUQA-111	240 (100%)	-	-	*Ehrlichia* sp. EHh317
WHCUQA-97	1300 (99.8%)	1000 (100%)	700 (99.6%)	*R. monacensis*
WHCUQA-63	240 (98.7%)	-	-	*C.* N. mik
WHCUQA-LabF56	650 (100%)	-	-	*R. japonica*
WHCUQA-LabP13	650 (99.5%)	-	-	*R. japonica*
WHCUQA-LabP36	650 (99.5%)	-	-	*R. japonica*
WHCUQA-LabP43	650 (99.5%)	-	-	*R. japonica*
WHCUQA-LabP48	650 (99.5%)	-	-	*R. japonica*
WHCUQA-LabP88	650 (100%)	-	-	*R. japonica*
Culex tritaeniorhynchus				
Egg				
WHCUTE-LabF3	650 (100%)	-	-	*R. sibirica*
WHCUTE-LabF5	650 (99.8%)	-	-	*R. monacensis*
Larvae				
WHCUTL-97	650 (99.2%)	-	-	*A. platys*
WHCUTL-131	650 (98.3%)	-	-	*A. platys*
WHCUTL-65	650 (97.6%)	-	-	*C.* R. ct
WHCUTL-121	240 (98.7%)	-	-	*C.* N. mik
WHCUTL-LabF11	650 (100%)	-	-	*R. sibirica*

(*Continued*)

Strains	Genes(nt)			Bacteria
	rrs	*gltA*	*groEL*	
WHCUTL-LabF24	650 (99.8%)	-	-	*R. monacensis*
WHCUTL-LabF28	1100 (100%)	-	-	*R. sibirica*
WHCUTL-LabF41	650 (100%)	-	-	*R. japonica*
WHCUTL-LabF43	650 (100%)	-	-	*R. japonica*
WHCUTL-LabF80	1100 (99.9%)	-	-	*R. monacensis*
Pupae				
WHCUTP-70	240 (100%)	-	-	*E. chaffeensis*
WHCUTP-78	240 (98.7%)	-	-	*C.* N. mik
WHCUTP-122	240 (98.7%)	-	-	*C.* N. mik
WHCUTP-LabF7	650 (100%)	-	-	*R. sibirica*
WHCUTP-LabF9	1100 (99.8%)	-	-	*R. monacensis*
WHCUTP-LabF18	1100 (99.9%)	-	-	*R. monacensis*
WHCUTP-LabF23	650 (99.8%)	-	-	*R. monacensis*
WHCUTP-LabF33	1100 (100%)	-	-	*R. japonica*
WHCUTP-LabF61	1100 (99.8%)	-	-	*R. monacensis*
WHCUTP-LabF64	650 (99.8%)	-	-	*R. monacensis*
WHCUTP-LabF94	1100 (99.8%)	-	-	*R. monacensis*
WHCUTP-LabF97	1100 (99.9%)	-	-	*R. monacensis*
WHCUTP-LabF110	1100 (99.8%)	-	-	*R. monacensis*
WHCUTP-LabF112	1100 (99.9%)	-	-	*R. monacensis*
WHCUTP-LabF118	650 (99.8%)	-	-	*R. monacensis*
Adult				
WHCUTA-25	1381 (99.6%)	-	-	*A. marginale*
WHCUTA-8	1381 (99.4%)	-	-	*A. marginale*
ZJCUTA-10	1370 (99.6%)	-	-	*A. marginale*
ZJCUTA-2	1379 (99.3%)	-	-	*A. marginale*
ZJCUTA-30	1351 (99.8%)	-	-	*A. marginale*
ZJCUTA-5	1380 (99.6%)	-	-	*A. marginale*
ZJCUTA-6	1380 (99.7%)	-	-	*A. marginale*
WHCUTA-99	650 (99.1%)	-	-	*A. phagocytophilum*
WHCUTA-111	650 (99.2%)	-	-	*A. platys*
WHCUTA-122	650 (99.4%)	-	-	*A. platys*
WHCUTA-123	650 (98.6%)	-	-	*A. platys*
WHCUTA-137	650 (99.4%)	-	-	*A. platys*
WHCUTA-141	650 (98.5%)	-	-	*A. platys*
WHCUTA-121	1300 (99.3%)	1000 (76.7%)	-	R. nc
WHCUTA-130	1300 (99.3%)	1000 (76.7%)	-	R. nc
WHCUTA-LabF5	650 (100%)	-	-	*R. japonica*
WHCUTA-LabF11	1100 (100%)	-	-	*R. japonica*

(*Continued*)

**Strains**	**Genes(nt)**			**Bacteria**
	rrs	*gltA*	*groEL*	
WHCUTA-LabF12	1100 (99.9%)	-	-	*R. japonica*
WHCUTA-LabF13	650 (99.7%)	-	-	*R. sibirica*
WHCUTA-LabF15	1100 (100%)	-	-	*R. japonica*
WHCUTA-LabF20	1100 (99.7%)	-	-	*R. monacensis*
WHCUTA-LabF33	1100 (100%)	-	-	*R. japonica*
WHCUTA-LabF34	1100 (100%)	-	-	*R. japonica*
WHCUTA-LabF35	650 (99.7%)	-	-	*R. sibirica*
WHCUTA-LabF42	1100 (100%)	-	-	*R. japonica*
WHCUTA-LabF46	650 (99.7%)	-	-	*R. sibirica*
WHCUTA-LabF47	650 (100%)	-	-	*R. japonica*
WHCUTA-LabF51	650 (100%)	-	-	*R. japonica*
WHCUTA-LabF53	650 (100%)	-	-	*R. japonica*
WHCUTA-LabF54	1100 (99.9%)	-	-	*R. japonica*
WHCUTA-LabF59	650 (99.7%)	-	-	*R. sibirica*
WHCUTA-LabF63	1100 (99.8%)	-	-	*R. japonica*
WHCUTA-LabF64	1100 (99.8%)	-	-	*R. sibirica*
WHCUTA-LabF70	1100 (99.7%)	-	-	*R. sibirica*
WHCUTA-LabF71	650 (100%)	-	-	*R. japonica*
WHCUTA-LabF84	650 (100%)	-	-	*R. japonica*
WHCUTA-LabF91	650 (100%)	-	-	*R. japonica*
WHCUTA-LabP31	650 (100%)	-	-	*R. japonica*
WHCUTA-LabP52	650 (100%)	-	-	*R. japonica*
WHCUTA-LabP114	650 (99.6%)	-	-	*R. japonica*
WHCUTA-LabP157	650 (99.5%)	-	-	*R. monacensis*
WHCUTA-LabP162	650 (100%)	-	-	*R. sibirica*

[a] The abbreviation is the same as in Tables 1 and 3.

[b] Nucleotide sequence identity compared to the reference sequences from GenBank.

[c] “-”, not available.

Supplementary Table S2. Prevalence of Rickettsiales in adult mosquito sampled from three regions of China.

Species	Location	Years	*Anaplasma*[a]						*Ehrlichia*			*Rickettsia*				*C.N.mik*	Total
			A.bov	*C.*A.bol	*A.mar*	*A.pha*	*A.pla*	*C.*A.rod	*E.cha*	E.ehh	E.ns	*C.*R.as	*R.bel*	*R.mon*	R.nc		
Ae.a.	Hubei	2015	0	0	0	0	0	0	0	0	0	0	0	0	0	1	1/144
	Zhejiang	2014	0	1	2	1	0	0	0	0	0	0	0	0	0	0	4/6
An.s.	Hubei	2014	5	1	25	0	8	0	0	0	0	0	0	0	0	0	39/96
		2015	0	0	0	0	0	2	0	1	0	1	1	0	0	2	7/96
	Jiangxi	2014	2	0	4	0	2	0	0	0	0	0	0	0	0	0	8/38
	Zhejiang	2014	0	0	1	0	0	0	0	0	0	0	0	0	0	0	1/48
Ar.s.	Hubei	2014	2	0	4	1	3	0	0	0	0	0	0	0	0	0	10/48
		2015	3	0	0	3	0	1	0	2	1	0	0	0	0	7	17/55
	Jiangxi	2014	0	2	0	4	1	0	0	0	0	0	0	0	0	0	7/42
	Zhejiang	2014	0	1	0	1	2	0	0	0	0	0	0	0	0	0	4/14
Cu.q.	Hubei	2014	0	0	0	0	0	0	0	0	0	0	0	0	0	0	0/48
		2015	0	0	0	0	0	2	1	2	0	0	0	1	0	1	7/96
Cu.t.	Hubei	2014	0	0	2	0	0	0	0	0	0	0	0	0	0	0	2/48
		2015	0	0	0	1	5	0	0	0	0	0	0	0	2	0	8/96
	Zhejiang	2014	0	0	5	0	0	0	0	0	0	0	0	0	0	0	5/96
Total			12	5	43	11	21	5	1	5	1	1	1	1	2	11	120/971

[a] Abbreviations are the same as those in Table 1.

Supplementary Table S3. Prevalence of Rickettsiales in eggs, larvae and pupae sampled from Hubei Province, China.

Species	Years	*Anaplasma*[a]						*Ehrlichia*			*Rickettsia*	*C.*N.mik	Total
		A.bov	*C.*A.bol	*A.pla*	*A.mar*	*A.pha*	*C.*A.rod	*E.cha*	E.ehh	E.ns	*C.*R.ct		
Egg													
Ae.a	2014	0	0	0	0	0	0	0	0	0	0	0	0/0
	2015	0	0	0	0	0	0	0	0	0	0	0	0/0
An.s	2014	0	0	0	1	1	0	0	0	0	0	0	2/4
	2015	0	0	0	0	0	0	0	0	0	0	0	0/5
Ar.s	2014	0	0	0	0	0	0	0	0	0	0	0	0/0
	2015	0	0	0	0	0	0	0	0	0	0	0	0/0
Cu.q	2014	0	0	0	0	0	0	0	0	0	0	0	0/0
	2015	0	0	0	0	0	0	0	0	0	0	0	0/0
Cu.t	2014	0	0	0	0	0	0	0	0	0	0	0	0/10
	2015	0	0	0	0	0	0	0	0	0	0	0	0/0
Subtotal		0	0	0	1	1	0	0	0	0	0	0	2/19
Larvae													
Ae.a	2014	1	1	1	2	5	0	0	0	0	0	0	10/48
	2015	3	0	0	2	2	1	0	6	1	0	2	17/96
An.s	2014	2	0	1	5	5	0	0	0	0	0	0	13/48
	2015	0	0	0	0	0	0	0	0	0	0	0	0/40
Ar.s	2014	6	1	2	6	10	0	0	0	0	0	0	25/48
Cu.q	2014	0	1	0	0	3	0	0	0	0	0	0	4/48
	2015	0	0	0	2	0	2	1	0	0	0	2	7/96
Cu.t	2014	0	0	0	0	0	0	0	0	0	0	0	0/96
	2015	0	0	2	0	0	0	0	0	0	1	1	4/48
Subtotal		12	3	6	17	25	3	1	6	1	1	5	80/528
Pupae													
Ae.a	2014	0	0	1	1	0	0	0	0	0	0	0	2/48
	2015	0	0	0	0	0	1	0	2	0	0	2	5/96
An.s	2014	0	1	1	1	0	0	0	0	0	0	0	3/48
	2015	0	0	0	0	0	0	0	0	0	0	1	1/40
Ar.s	2014	2	1	1	2	0	0	0	0	0	0	0	6/48
Cu.q	2015	1	0	0	0	0	0	0	1	0	0	1	3/144
Cu.t	2014	0	0	0	0	0	0	0	0	0	0	0	0/48
	2015	0	0	0	0	0	0	1	0	0	0	2	3/82
Subtotal		3	2	3	4	0	1	1	3	0	0	6	23/554

[a] Abbreviations are the same as those in Tables 1 and 3.

Supplementary Table S4. Primers used in this study.

Bacteria	gene	Sequences (5'→3')	Position[a]	Reference
Rickettsiales	***rrs***	Eh-out1: TTGAGAGTTTGATCCTGGCTCAGAACG(+)	1-27	26
		Eh-out2: CACCTCTACACTAGGAATTCCGCTATC(-)	653-627	26
		Eh-gs1: GTAATAACTGTATAATCCCTG(+)	167-187	26
		Eh-gs2: GTACCGTCATTATCTTCCCTA(-)	448-428	26
Anaplasma	***rrs***	GTTTGATCCTGGCTCAGAA (+)	15-33	This study
		TACACTAGGAATTCCGCTATC (-)	652-632	This study
		CTAATACCGTATATGCYC (+)	173-190	This study
		GTACCGTCATTATCWTCC (-)	453-436	This study
Anaplasma	***rrs***	GGATAGCCACTRGAARTGGTG(+)	151-171	This study
		CGTGCTGACTTGACATCAT(-)	1172-1154	This study
		CATCTCACGACACGAGCTG(-)	1049-1031	This study
		CTGTCTGGTCCGGTACTGAC(+)	700-719	This study
		TGGTCCGGTACTGACRCT(+)	705-722	This study
		TGCCTCCTTDCGGTTGGC(-)	1425-1408	This study
Ehrlichia	***rrs***	GAATAGCCATTAGAAATGATG(+)	155-175	This study
		GTCAGTATCGAACCAGATAG(-)	723-704	This study
		GTATCGAACCAGATAGCCG(-)	719-701	This study
		CGGCTATCTGGTTCGATAC(+)	701-719	This study
		CTATCTGGTTCGATACTGAC(+)	704-723	This study
		GCTTCCTTKCGGTTAGCAC(-)	1427-1409	This study
Rickettsia	***rrs***	GTACGGAATAACTTTTAGAAAT(+)	147-168	This study
		CATGATGACTTGACRTCGT(-)	1175-1157	This study
		CATCTCACGACACGAGCTG(-)	1052-1034	This study
		GAAGGCGRTCATYTRGGCT(+)	693-711	This study
		GRTCATYTRGGCTRCAACTG(+)	699-718	This study
		CTGCCTCTTGCGTTAGCT(-)	1427-1410	This study
Ca. Neoehrlichia	***rrs***	GACAGGTAATACCRTATAATCCCT(+)	123-146	This study
		GTCAGAACTGAGCCAGATAG(-)	675-656	This study
		CTGAGCCAGATAGTCGC(-)	668-652	This study
		GCGACTATCTGGCTCAG(+)	652-668	This study
		CTATCTGGCTCAGTTCTGAC(+)	656-675	This study
		TGCCTCCTTACGGTTAG(-)	1382-1366	This study
A. platys	***groEL***	AGTCGATTAGGGAAGTAGTAC(+)	38-58	This study
		AGGATGGCTACAAGGTAATG(+)	149-168	This study
		GCGTCCTCTACTCTGTCTT(-)	1199-1181	This study
A. marginale	***groEL***	ACATGCTCCATACTGACTGC(+)	268-287	This study
		AGATGAGATTGCACAGGTTG(+)	420-439	This study
		AGATGCAAGCGTGTATAGCAG(-)	1281-1261	This study
A. bovis	***groEL***	GCTGCTGCGATAGCTAATAT(+)	196-215	This study
		GATGGTACCACTACTTGTTCA(+)	256-276	This study
		CTATGACTGCTATGTCACCA(-)	883-864	This study

(*Continued*)

Bacteria	Genes	Sequences (5'→3')	Position	Reference
Ca. A. boleense	***groEL***	TAGAAGACGCGGTAGGCT(+)	47-64	This study
		GTACTGCAGGCCCTAAAG(+)	65-82	This study
		AACGTTCTCCAATATGGGAAG(-)	679-699	This study
Ehrlichia sp. EHh317	***groEL***	GAAGATGCTGTAGGRTGTACDGC(+)	67-89	This study
		ATTRCTCARAGTGCTTCHCARTG(+)	217-239	This study
		AGHGCTTCWCCTTCYACATCYTC(-)	776-754	This study
Rickettsia	***groEL***	CCATTACATGATAGAATTGCAAT(+)	***groES***	This study
		GAATTGCAATAAAGCCTATCG(+)	***groES***	This study
		CCATCATTGCTTTTCTTCTATC(-)	868-847	This study
A. platys	***gltA***	TGRAAGAAAAWGCTGTTTTG(+)	2-21	This study
		AGCTRTTTTRGAGTGYGGAG(+)	12-31	This study
		GCTCTRGGRTCATARCTYTT(-)	926-907	This study
A. mraginale	***gltA***	TGGTAGAAAAAGCGATTTTAG(+)	2-22	This study
		ATAAGCTTGCCCGTTATGC(+)	40-48	This study
		CCGGTATAAAGTTGGCGT(-)	1235-1218	This study
Ca. A. boleense	***gltA***	ATGTCTACTGCRGCWTGC(+)	1-18	This study
		TACTGCRGCTWGCAGGTCT (+)	6-24	This study
		CAGYAGTTCTMGCTAATG(-)	1012-996	This study
Rickettsia	***gltA***	CCGGGYTTTATGTCTACTGC(+)	151-170	This study
		CTTTATGTCTACTGCKTCTTG(+)	156-176	This study
		AGCTGTCTWGGTCTGCTGATT(-)	1259-1239	This study

[a] The numbers correspond to genome positions of reference sequences from GenBank.

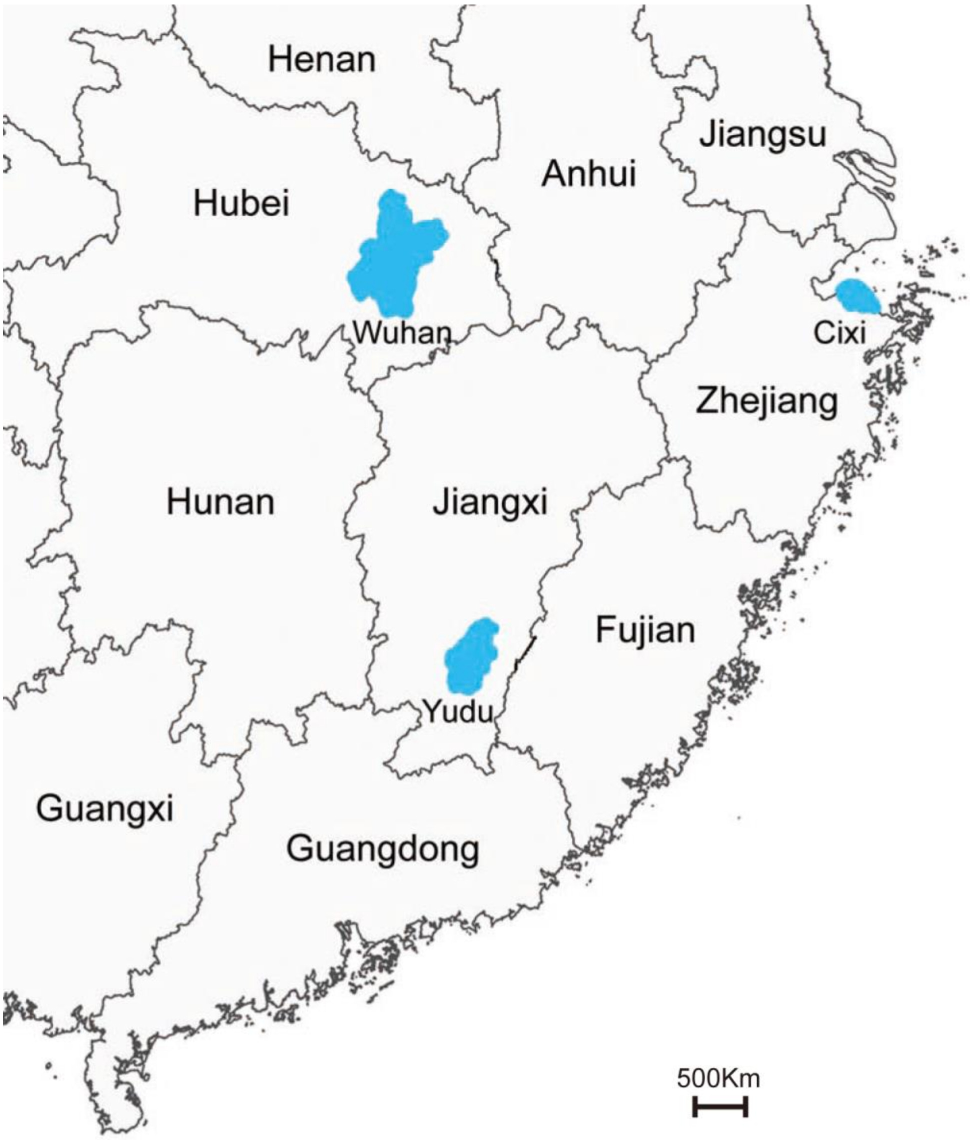

Supplementary Figure S1. Map showing the location of the collection areas in Hubei, Jiangxi, and Zhejiang provinces, China. The areas marked with blue represented the locations of Wuhan, Cixi and Yudu, respectively. The figure was generated using ArcGIS (version 10.0; http://www.arcgis.com/feature/).
